# A Comparative Study of the Bias Correction Methods for Differential Item Functioning Analysis in Logistic Regression with Rare Events Data

**DOI:** 10.1155/2020/1632350

**Published:** 2020-02-25

**Authors:** Marjan Faghih, Zahra Bagheri, Dejan Stevanovic, Seyyed Mohhamad Taghi Ayatollahi, Peyman Jafari

**Affiliations:** ^1^Department of Biostatistics, Faculty of Medicine, Shiraz University of Medical Sciences, Shiraz, Iran; ^2^Clinic for Neurology and Psychiatry for Children and Youth, Belgrade, Serbia

## Abstract

The logistic regression (LR) model for assessing differential item functioning (DIF) is highly dependent on the asymptotic sampling distributions. However, for rare events data, the maximum likelihood estimation method may be biased and the asymptotic distributions may not be reliable. In this study, the performance of the regular maximum likelihood (ML) estimation is compared with two bias correction methods including weighted logistic regression (WLR) and Firth's penalized maximum likelihood (PML) to assess DIF for imbalanced or rare events data. The power and type I error rate of the LR model for detecting DIF were investigated under different combinations of sample size, moderate and severe magnitudes of uniform DIF (DIF = 0.4 and 0.8), sample size ratio, number of items, and the imbalanced degree (*τ*). Indeed, as compared with WLR and for severe imbalanced degree (*τ* = 0.069), there were reductions of approximately 30% and 24% under DIF = 0.4 and 27% and 23% under DIF = 0.8 in the power of the PML and ML, respectively. The present study revealed that the WLR outperforms both the ML and PML estimation methods when logistic regression is used to evaluate DIF for imbalanced or rare events data.

## 1. Introduction

In psychological and educational tests, measurement invariance is a crucial assumption for comparison of mean scores across people from different cultural, racial, or demographic backgrounds. Assessing this statistical property at the item level, also known as differential item functioning (DIF), is an important part of the process of validating tests. In general, DIF analysis is used to distinguish whether the probability of responding to a specific item on a multi-item scale differs between two groups after controlling for the overall ability that is being measured by the a questionnaire [1]. Nowadays, different statistical methods including the logistic regression (LR) model, multiple group confirmatory factor analysis (MGCFA), and item response theory (IRT) model are available to assess the presence of DIF among subgroups of people [2, 3]. The LR is a model-based approach first introduced by Swaminathan and Rogers to assess DIF for both dichotomous and polytomous item scored [4, 5]. The LR model is able to control additional continuous and categorical confounders which may affect the results of DIF analysis. Furthermore, the LR model provides a number of effect size measures to quantify the magnitude of uniform and nonuniform DIF which may not be practically or clinically important [6–8]. Uniform DIF occurs when the difference in item response probabilities is constant across the scale. Nonuniform DIF is evident when the direction of DIF differs in different parts of the construct scale [9, 10]. Previous simulation studies have shown that identification of DIF through the LR model may be affected by various factors such as sample size, sample size ratio, magnitude of DIF, scale length (the number of items), and the number of groups [11–14].

However, it should be noted that statistical inference based on the logistic regression model is highly dependent on the asymptotic properties of the maximum likelihood estimator [9]. Under the large sample situations, the sampling distribution of the maximum likelihood (ML) estimators for the logistic regression coefficients is asymptotically unbiased and normal. However, in small samples, it is well known that the ML estimations may be biased and the asymptotic properties may not hold [9, 15]. Accordingly, a number of penalization techniques such as Firth's method have been introduced to correct or reduce the small sample bias of the ML estimators of the LR model [15, 16]. A new simulation study has recently compared the performance of the LR model based on maximum likelihood (ML) and Firth's penalized maximum likelihood (PML) estimation methods in terms of empirical power and type I error rate to detect uniform and nonuniform DIF [9]. The results showed that, as compared with PML, the LR model based on asymptotic ML worked slightly better in terms of statistical power although the difference in performance was not practically important [9].

In addition to the small sample, rare events data is another factor that can substantially influence the asymptotic properties of the ML estimators in the LR model [15, 17]. The bias of prediction, as well as bias in regression coefficients, is a potential problem that may arise from the use of standard logistic regression in the presence of rare events data. Rare events data refer to occurrences that take place much less frequently than more common events [18, 19]. According to King and Zeng, ordinary logistic regression can sharply underestimate the probability of rare events [20]. Hence, they proposed weighted logistic regression (WLR) to correct bias terms in regression coefficients and predicted probability by applying a weighted likelihood function to estimate parameters under pure case-control sampling [20]. Moreover, a wide variety of penalization methods have been suggested to resolve the problem of rare events data in the LR model [15–17].

Although the effect of bias correction methods on the performance of the LR model for detecting DIF has been evaluated by Lee in a small sample [9], such an explanation has never been provided for rare events data. To fill this gap, in the present simulation study, the three inferential methods including WLR, PML, and ML are compared to discover what the best correction method is to achieve the adequate power and type I error rate for detecting DIF when the response variable in the LR model is imbalanced or rare. Hence, in a comprehensive simulation study, we investigate whether the statistical properties of the LR model for detecting DIF, with and without applying bias correction methods, can be influenced by the degree of rareness or imbalance data, magnitude of DIF, sample size, sample size ratio, and the length of the scale across reference and focal groups. In addition to simulation, we have also used real data to validate and compare the effectiveness of the proposed methods for detecting DIF in practice.

## 2. Methods

### 2.1. Logistic Regression Model for Detecting DIF

The presence of uniform and nonuniform DIF can be tested by comparing three different logistic regression (LR) models as follows:

(1) $$\begin{array}{c} \text{Model }1:\;\log\text{ it}\left(\pi_{i}\right)=\ln\left(\frac{p\left(y_{i}=1\right)}{p\left(y_{i}=0\right)}\right)=\beta_{1}+\beta_{2}\theta ,\\ \text{Model }2:\;\log\text{ it}\left(\pi_{i}\right)=\ln\left(\frac{p\left(y_{i}=1\right)}{p\left(y_{i}=0\right)}\right)=\beta_{1}+\beta_{2}\theta +\beta_{3}\text{G,}\\ \text{Model }3:\;\log\text{ it}\left(\pi_{i}\right)=\ln\left(\frac{p\left(y_{i}=1\right)}{p\left(y_{i}=0\right)}\right)=\beta_{1}+\beta_{2}\theta +\beta_{3}G+\beta_{4}\theta G. \end{array}$$

In these models, the term *θ* is the observed ability of each respondent usually defined as total test score, and *G* is an indicator variable representing group membership (reference and focal groups, for example, male and female in gender variable). According to the abovementioned models, the presence of uniform and nonuniform DIF could be determined by comparing models 1 and 2 as well as models 2 and 3, respectively. In both cases, the difference between −2 log likelihoods of the models is compared to a *χ*^2^ distribution with one degree of freedom.

For nonuniform DIF items, the direction of DIF differs across latent constructs, and the effect of DIF is naturally cancelled out at the level of latent constructs [21] which is the major reason for focusing on uniform DIF in this study.

### 2.2. Maximum Likelihood

Consider the logistic regression model

(2) $$\begin{array}{c} P\left(\left.y_{i}=1\right|x_{ir},B\right)=\pi_{i}=\frac{e^{X_{i}\beta }}{1+e^{X_{i}\beta }}=\frac{1}{1+e^{-X_{i}\beta }}=\frac{1}{1+\exp\left(-\sum_{r=1}^{k}\beta_{r}x_{ir}\right)},\\ \text{so},\;\ln\left(\frac{\pi_{i}}{1-\pi_{i}}\right)=X_{i}\beta , \end{array}$$

where (*y*_*i*_, *x*_*ir*_), *x*_*ir*_ (*i* = 1,…, *n*; *r* = 2,…, *k*) and *y*_*i*_ ∈ {0, 1}, denotes a sample of *n* observations of binary outcome variable *y* and the vector of independent covariates with 1 × *k* dimensions  **X**_*i*_=(1, *x*_*i*2_,…, *x*_*ik*_), where *x*_*i*1_ = 1 is the constant and **β**=(*β*_1_,…,*β*_*k*_)′. Then, ML estimates ${\hat{\beta }}_{r}$ of regression parameters, and *β*_*r*_ are obtained by solving the following score equations:

(3) $$\begin{array}{c} \frac{\partial \;\ln\;L}{\partial \beta_{r}}\equiv U\left(\beta_{r}\right)=\sum_{i=1}^{n}\left(y_{i}-\pi_{i}\right)x_{ir}=0, \end{array}$$

where

(4) $$\begin{array}{c} L\left(\beta \right)=\prod_{i=1}^{n}{\left(\pi_{i}\right)}^{y_{i}}{\left(1-\pi_{i}\right)}^{1-y_{i}}=\prod_{i=1}^{n}{\left(\frac{e^{X_{i}\beta }}{1+e^{X_{i}\beta }}\right)}^{y_{i}}{\left(\frac{1}{1+e^{X_{i}\beta }}\right)}^{1-y_{i}},\\ \ln\;\;L\left(\beta \right)=\sum_{i=1}^{n}\left(y_{i}\;\;\ln\;\pi_{i}+\left(1-y_{i}\right)\ln\left(1-\pi_{i}\right)\right)=\sum_{i=1}^{n}\left(y_{i}\;\;\ln\left(\frac{e^{X_{i}\beta }}{1+e^{X_{i}\beta }}\right)+\left(1-y_{i}\right)\ln\left(\frac{1}{1+e^{X_{i}\beta }}\right)\right). \end{array}$$

Unfortunately, there is no closed-form expression for maximizing the log likelihood of *β*. The ML estimations can, therefore, be obtained using numerical optimization methods, which start with a guess point and repeat to improve. The Newton–Raphson method is one of the most commonly used numerical methods, which needs the Hessian matrix as follows:

(5) $$\begin{array}{c} \frac{\partial^{2}\ln\;L}{\partial \beta_{r}{}^{2}}=\sum_{i=1}^{n}\left(\frac{-x_{ir}^{2}e^{X_{i}\beta }}{{\left(1+e^{X_{i}\beta }\right)}^{2}}\right)=\sum_{i=1}^{n}\left(-x_{ir}{}^{2}\left(\pi_{i}\left(1-\pi_{i}\right)\right)\right). \end{array}$$

If *v*_*i*_ is defined as *π*_*i*_(1 − *π*_*i*_) and  *V*=diag(*v*_1_,…, *v*_*n*_), then the Hessian matrix can be written as

(6) $$\begin{array}{c} H\left(\beta \right)=-X^{\prime }VX. \end{array}$$

The information matrix is given by *I*(*β*)=−(∂^2^ln*L*/∂*β*^2^)=*X*′*VX*, and the variance of *β* is then *V*(*β*)=*I*(*β*)^−1^=(*X*′*VX*)^−1^.

### 2.3. Penalized Maximum Likelihood

The penalized maximum likelihood estimation method (PML) was originally developed by David Firth [16] in order to reduce the small sample bias of maximum likelihood estimates. For exponential family models, this method corresponds to penalization of the likelihood by Jeffreys' invariant prior [22]. Thus, the penalized log likelihood for logistic regression takes the following form:

(7) $$\begin{array}{c} \ln\;L{\left(\beta \right)}^{*}=\ln\;L\left(\beta \right)+.5\;\ln\left|I\left(\beta \right)\right|, \end{array}$$

where  |*I*(*β*)| denotes the determinant of the Fisher information matrix evaluated at *β*. Penalized maximum likelihood estimates for *β*_*r*_ (*r* = 1,…, *k*) are involved in calculating

(8) $$\begin{array}{c} \frac{\partial \;\ln\;L{\left(\beta \right)}^{*}}{\partial \beta_{r}}=U{\left(\beta_{r}\right)}^{*}=\overset{n}{\underset{i=1}{\sum }}\left(y_{i}-\pi_{i}+h_{i}\left(0.5-\pi_{i}\right)\right)x_{ir}=0,\;r=1,\ldots ,k, \end{array}$$

where the *h*_*i*_'s represent the diagonal elements of the penalized likelihood version of the standard “hat” matrix:

(9) $$\begin{array}{c} H=V^{1/2}X{\left(X^{\prime }VX\right)}^{-1}X^{\prime }V^{1/2}. \end{array}$$

By this method, the first-order term is removed from the Taylor series expansion of the bias of the ML estimator, which has negligible impacts in large samples but can be severe in small samples or rare events.

Now, Firth-type estimation, $\hat{\beta }$, can be produced iteratively by Newton–Raphson algorithm with a starting value of ${\hat{\beta }}^{\left(0\right)}=0$ until convergence is obtained.

(10) $$\begin{array}{c} {\hat{\beta }}^{\left(s+1\right)}={\hat{\beta }}^{s}+I^{-1}\left({\hat{\beta }}^{s}\right)U{\left({\hat{\beta }}^{s}\right)}^{*}, \end{array}$$

where the superscript *s* is the *s*th iteration. If the penalized log likelihood assessed at ${\hat{\beta }}^{\left(s+1\right)}$ is less than that assessed at ${\hat{\beta }}^{s}$, then ${\hat{\beta }}^{\left(s+1\right)}$ is recalculated by step-halving. The PML estimations can also be created by carrying out the MLE method and splitting each main observation *i* into two new observations having outcomes, 1 − *y*_*i*_ and *y*_*i*_, with weights *h*_*i*_/2 and 1+(*h*_*i*_/2), respectively. The new observations change the score function for the ML method to {(*y*_*i*_ − *π*_*i*_)(1+(*h*_*i*_/2))+(1 − *y*_*i*_ − *π*_*i*_)*h*_*i*_/2}*x*_*ir*_={ *y*_*i*_ − *π*_*i*_+*h*_*i*_(0.5 − *π*_*i*_)}*x*_*ir*_. Therefore, the breaking of each observation into a nonresponse and a response ensures that the finite PML estimates always exist [23, 24].

The standard error estimation is based on the root of the inverse diagonal elements of the Fisher information matrix, which is approximated by {−(∂^2 ^ln *L*^*∗*^/∂*β*^2^)}^−1^ [16].

### 2.4. Weighted Logistic Regression

The weighted logistic regression (WLR) introduced by King and Zeng [20, 25] is one of the bias correction methods which considers two correction steps. The first correction concerns the weights, to make up for the differences in the proportion of events in the sample and population. So, the following weighted log-likelihood should be maximized:

(11) $$\begin{array}{c} \ln\;L_{\text{w}}\left(\left.\beta \right|y,X\right)=\sum_{i=1}^{n}w_{i}\ln\left(\frac{e^{y_{i}X_{i}\beta }}{1+e^{X_{i}\beta }}\right), \end{array}$$

where $w_{i}=w_{1}y_{i}+w_{0}\left(1-y_{i}\right),w_{0}=1-\tau /1-\bar{y},w_{1}=\tau /\bar{y}$, with $\bar{y}\;\text{and}\;\tau$ that are the fraction of 1s in the sample and population, respectively, in which the above weighted log-likelihood is obtained under pure case-control sampling as follows:

Suppose that the joint distribution of *y* and *X* in the sample is

(12) $$\begin{array}{c} f_{s}\left(y,\left.X\right|\beta \right)=P_{s}\left(\left.X\right|y,\beta \right)P_{s}\left(y\right). \end{array}$$

Yet, since *X* is a matrix of exploratory variables, then *P*_*s*_(**X**|**y**, **β**)=*P*(**X**|**y**, **β**). In the other words, the conditional probabilities of *X* in the sample and population are equal. However, the conditional probability of the population is

(13) $$\begin{array}{c} P\left(\left.X\right|y,\beta \right)=\frac{f\left(y,\left.X\right|\beta \right)}{P\left(y\right)}. \end{array}$$

But,

(14) $$\begin{array}{c} f\left(y,\left.X\right|\beta \right)=P\left(\left.y\right|X,\beta \right)P\left(X\right). \end{array}$$

Also, by replacing and rearranging,

(15) $$\begin{array}{c} f_{s}\left(y,\left.X\right|\beta \right)=P_{s}\left(\left.X\right|y,\beta \right)P_{s}\left(y\right)=P\left(\left.X\right|y,\beta \right)P_{s}\left(y\right)=\frac{f\left(y,\left.X\right|\beta \right)}{\text{P}\left(y\right)}P_{s}\left(y\right)=\frac{P_{s}\left(y\right)}{P\left(y\right)}P\left(\left.y\right|X,\beta \right)P\left(X\right)=\frac{\text{H}}{\text{Q}}P\left(\left.y\right|X,\beta \right)P\left(X\right), \end{array}$$

where *H* and *Q* represent the proportions in the sample and population, respectively. The likelihood is then

(16) $$\begin{array}{c} L\left(\beta \right)=\prod_{i=1}^{n}\frac{H_{i}}{Q_{i}}\;P\left(\left.y_{i}\right|x_{i},\beta \right)P\left(x_{i}\right). \end{array}$$

The log-likelihood for WLR can then be rewritten as

(17) $$\begin{array}{c} \ln\;L_{w}\left(\left.\beta \right|y,X\right)=\sum_{i=1}^{n}\frac{Q_{i}}{H_{i}}\ln\;P\left(\left.y_{i}\right|x_{i},\beta \right)=\sum_{i=1}^{n}\frac{Q_{i}}{H_{i}}\ln\left(\frac{e^{y_{i}X_{i}\beta }}{1+e^{X_{i}\beta }}\right)=\sum_{i=1}^{n}w_{i\;}\ln\left(\frac{e^{y_{i}X_{i}\beta }}{1+e^{X_{i}\beta }}\right), \end{array}$$

where *w*_*i* _=*Q*_*i*_/*H*_*i*_. Thus, the likelihood function must be multiplied by the inverse of the fractions in order to obtain a consistent estimator. If the proportion of events in the population is more than that in the sample, then *w*_*i* _ is more than one, and so the nonevent are given less weight, and vice versa [26, 27]. The weighting method has two serious problems that limit its application: first, the calculation of the standard errors through the information matrix is severely biased. This problem will be solved by using White's heteroscedasticity-consistent variance matrix [20].

Second, slope coefficient is biased in rare events data. For solving the second problem, the bias in $\hat{\beta }$ can be estimated by the following weighted least-squares expression:

(18) $$\begin{array}{c} \text{bias}\left(\hat{\beta }\right)={\left(X^{\prime }VX\right)}^{-1}X^{\prime }V\xi . \end{array}$$

If bias ($\hat{\beta }$) is the bias component and $\hat{\beta }$ is the uncorrected coefficient, then the corrected coefficients $\tilde{\beta }$ is $\tilde{\beta }=\hat{\beta }-\text{bias}\left(\hat{\beta }\right)$, where

(19) $$\begin{array}{c} \xi_{i}=0.5\;Q_{ii}\left[\left(1+w_{1}\right){\hat{\pi }}_{i}-w_{1}\right]. \end{array}$$

Also, *Q*_*ii*_ are the diagonal elements of *Q*=*X*(*X*′*VX*)^−1^*X*′, and $V=\text{diag}\left\{{\hat{\pi }}_{i}\left(1-{\hat{\pi }}_{i}\right)w_{i\;}\right\}$.

The variance matrix of $\tilde{\beta }$ is

(20) $$\begin{array}{c} V\left(\tilde{\beta }\right)={\left(\frac{n}{n+k}\right)}^{2}V\left(\hat{\beta }\right). \end{array}$$

Since (*n*/(*n*+*k*))^2^ < 1, then $V\left(\tilde{\beta }\right)<V\left(\hat{\beta }\right)$, and so both the variance and the bias are now diminished.

The second correction step is to adjust the underestimation of the probabilities when using the bias-corrected coefficients in the logistic model. By subtracting a correction factor *C*_*i*_ to the biased value of probability ${\tilde{\pi }}_{i}$,

(21) $$\begin{array}{c} \pi_{i}={\tilde{\pi }}_{i}-C_{i},\;C_{i}=\left(0.5-{\tilde{\pi }}_{i}\right){\tilde{\pi }}_{i}\left(1-{\tilde{\pi }}_{i}\right)X_{0}V\left(\tilde{\beta }\right)X_{0}^{\prime }, \end{array}$$

where $\;V\left(\tilde{\beta }\right)$ is the variance-covariance matrix, *X*_0_ is a vector of exploratory variables, and *π*_*i*_ is the approximate unbiased estimator by means of a known *τ*. When *τ* is unknown or partially known, King and Zeng introduced $\pi_{i}={\tilde{\pi }}_{i}+C_{i}$ as the approximate Bayesian estimator. In this case, the researcher may specify an upper and lower bound for the possible range of *τ*.

Maximum Likelihood (ML), Penalized Maximum Likelihood (PML), and rare event logistic regression were implemented using the “glm” function and the “logistf” R package, as well as the “relogit” function in the R package Zelig, respectively [28, 29].

### 2.5. Data Generation

In this study, an item response theory model for binary data was used to produce response data. The mathematical form of the IRT model is

(22) $$\begin{array}{c} p_{ij}\left(Y=\left.1\right|\theta_{i}\right)=\frac{e^{a_{j}\left(\theta_{i}-b_{j}\right)}}{1+e^{a_{j}\left(\theta_{i}-b_{j}\right)}}, \end{array}$$

where *p*_*ij*_(*θ*) is the probability of correct response for individual *i* of item *j*, *a*_*j*_ denotes the item discrimination parameter, *b*_*j*_ is the item difficulty parameter, and *θ*_*i*_ represents the ability level for the *i*th individual. In this study, *b*_*j*_ (*j* = 2, 3,…, *J*) and *θ* parameters were simulated from the standard normal distribution, and *a*_*j*_ (*j* = 2, 3,…, *J*) parameters were random samples of a uniform distribution within the interval (1, 2) [1].

In this simulation study, five factors were varied: sample size, magnitude of uniform DIF, sample size ratio, number of items, and the degree of rareness or imbalance. Three sample sizes (*N* = 200, 600, 1000) and three levels of sample size ratio (*R* = 1, 2, 3) were investigated. The sample size ratio between the focal and reference groups was set to 1 : 1 for the equal sample size conditions and 2 : 1 and 3 : 1 for the unequal sample size conditions. More specifically, we created conditions with *n*_R_/*n*_F_ = 100/100, 67/133, and 50/150 for the small sample size (*N* = 200) and *n*_R_/*n*_F_ = 300/300, 200/400, and 150/450 for the medium sample size (*N* = 600) and *n*_R_/*n*_F_ = 500/500, 333/667, and 250/750 for the large sample size (*N* = 1000). Furthermore, the two measures with 5 and 15 items were simulated (*I* = 5, 15).

To generate rare events data, we followed similar scenarios and notations used by King and Zeng. In order to generate imbalanced or rare events data, we applied the logit model: log it(*π*_*i*_)=*β*_0_+*β*_1_*x*_*i*_, *π*_*i*_=*p*(*Y*_*i*_=1), by holding *β*_1_ constant (*β*_1_ = 1) while varying *β*_0_. In this case, the values of *β*_0_ = −3 and *β*_0_ = −2 generate outcomes with the percentages of ones which are equal to 6.9% and 15.6%, respectively. It should be noted that we generate the simulated data based on the IRT model, not the logistic regression. Hence, we need to show that the parameters of the logistic regression and the IRT model with binary responses are one-to-one correspondent. In order to denote that both models have similar mathematical expressions, equation (22) can be rearranged as log it(*π*_*i*_)=−*a*_*j*_*b*_*j*_+*a*_*j*_*θ*_*i*_. By comparing this equation with the logit function log it(*π*_*i*_)=*β*_0_+*β*_1_*x*_*i*_, coefficients *β*_0_ and *β*_1_ and the variable *x*_*i*_ in the logistic regression model are found to be, respectively, correspondent to parameters −*a*_*j*_*b*_*j*_, *a*_*j*_ and *θ*_*i*_ in the IRT model (i.e. *β*_0_ = –*a*_*j*_*b*_*j*_, *β*_1_ = *a*_*j*_ and *x*_*i*_ = *θ*_*i*_). With the assumption of *a*_*j*_ = *β*_1_ = 1, the constant parameter (*β*_0_) in the logistic regression is equivalent to the minus value of the difficulty parameter (−*b*_*j*_) in the IRT model. Accordingly, in the IRT framework and when *a*_*j*_ = 1, with the values of *b*_*j*_ = 3 and 2, we can generate responses with 6.9% and 15.6% imbalanced degree, respectively.

Statistical power is defined by the proportion of times that DIF is correctly identified by the logistic method across replications, and the type I error rate, also referred to the false positive rate, represents the proportion of non-DIF items incorrectly flagged as having DIF in 1000 replications. The type I error rates are averaged over all without DIF items [30].

## 3. Results

Table 1 presents the statistical power of the LR model based on three different inferential methods (ML, PML, and WLR) and under various combinations of *τ*, *R*, *N*, DIF, and scale length. The major finding was that the power of the ML and PML was more affected by imbalanced degree (*τ*) than by WLR estimation method. Specifically, for the moderate magnitude of DIF (DIF = 0.4), regardless of the sample size and number of items, increasing imbalanced or rareness degree, *τ*, from 0.156 to 0.069 resulted in a reduction by approximately 38%, 37%, and 30% in the power of PML, ML, and WLR, respectively. Furthermore, for the severe magnitude of DIF (DIF = 0.8), a similar finding was observed, i.e., the power of the PML, ML, and WLR was reduced approximately by 37%, 35%, and 26%, respectively. In general, our findings indicated that the power of the three estimation methods for detecting DIF could be ordered from highest to lowest as follows: WLR ≥ ML ≥ PML. Indeed, for the moderate values of DIF (DIF = 0.4), compared with WLR, there were reductions of approximately 30% and 24% under *τ* = 0.069 and 22% and 16% under *τ* = 0.156 in the power for the PML and ML, respectively. On the other hand, when the magnitude of DIF was severe (DIF = 0.8), compared with WLR, there were reductions of approximately 27% and 23% under *τ* = 0.069 and 14% and 12% under *τ* = 0.156 in the power for the PML and ML, respectively.

When the magnitude of DIF was moderate (DIF = 0.4), *N* = 1000, *R* = 1 and *I* = 15, the maximum power of the PML, ML, and WLR was 46%, 48%, and 53% for *τ* = 0.156 and 25%, 28%, and 32% for *τ* = 0.069, respectively. Hence, to achieve the adequate power (0.8) for detecting moderate DIF, we require a sample size of larger than 1000 (*N* = 1000). Furthermore, for the severe of DIF (DIF = 0.8) and *τ* = 0.156, the power of the PML, ML, and WLR was 75%, 78%, and 83% for *I* = 5 and 80%, 81%, and 87% for *I* = 15, respectively, when *N* = 600 and *R* = 1, 2. However, under DIF = 0.8, *τ* = 0.069, *R* = 1, and *N* = 1000, the power of the PML, ML, and WLR was 66%, 69%, and 77% for *I* = 5 and 72%, 74%, and 81% for *I* = 15, respectively.

Table 2 reports the empirical type I error rate of the LR model under various combinations of imbalanced degree of data, sample size, sample size ratio, magnitude of DIF, and the number of items. In general, regardless of the magnitude of DIF, sample size, sample size ratio, and the number of items, the average type I error rates of the ML, PML, and WLR were 0.06, 0.05, and 0.03 for *τ* = 0.156 and 0.06, 0.04, and 0.01 for *τ* = 0.069. These findings indicate that when the imbalanced degree, *τ*, increased from 0.156 to 0.069, the average type I error rate of the WLR decreased dramatically, while it was close to the 5% level for the ML and PML methods. However, there were some exceptions where the type I error rates of the ML, PML, and WLR were higher than the nominal level of 5%. When DIF = 0.8, *N* = 1000, *I* = 5, and *τ* = 0.156, the empirical type I error rate was 11%, 11%, and 9% for ML, 8%, 9%, and 8% for PML and 8%, 7%, and 7% for WLR, when R was equal to 1, 2, and 3, respectively. In this case, as shown in Table 2, when the number of items increased from 5 to 15, the type I error rate would be equal or less than 5%.

**Figure 1:**
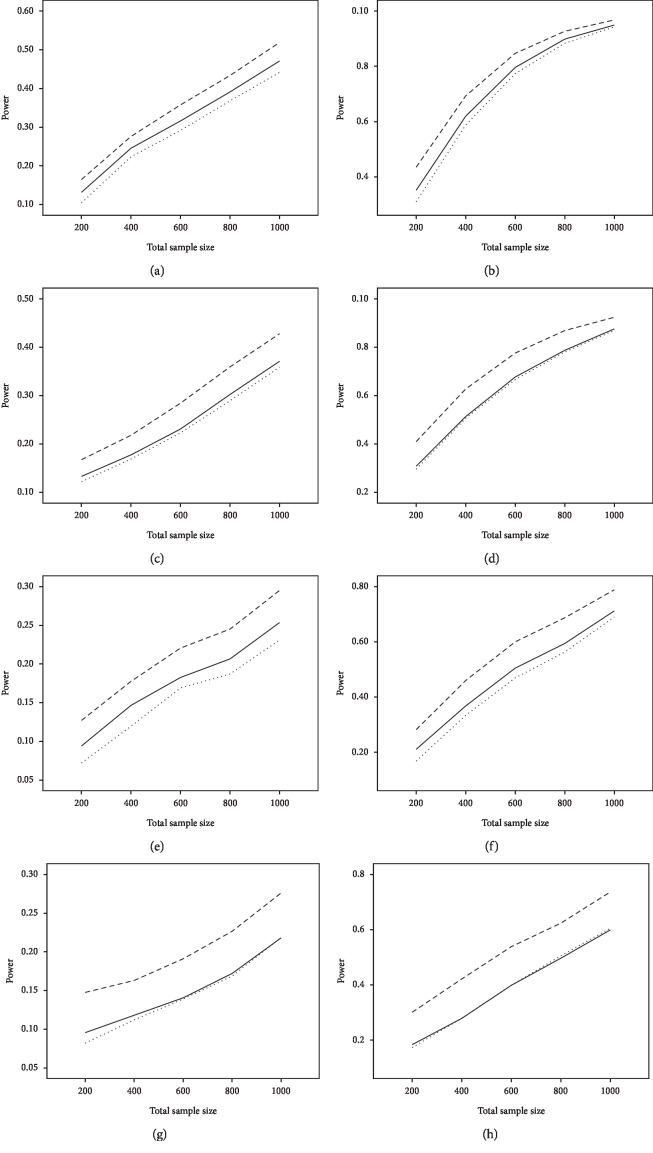
The average power of MLE (solid lines), PML (dotted line), and WLR (broken line) methods on measures with 5 and 15 items. *Note*. Left panel for DIF = 0.4 and right panel for DIF = 0.8. From top to bottom, the four panels are (*n*_f_ = *n*_r_, *τ* = 0.156), (*n*_f_ = 3*n*_r_, *τ* = 0.156), (*n*_f_ = *n*_r_, *τ* = 0.069), and (*n*_f_ = 3*n*_r_, *τ* = 0.069).

**Figure 2:**
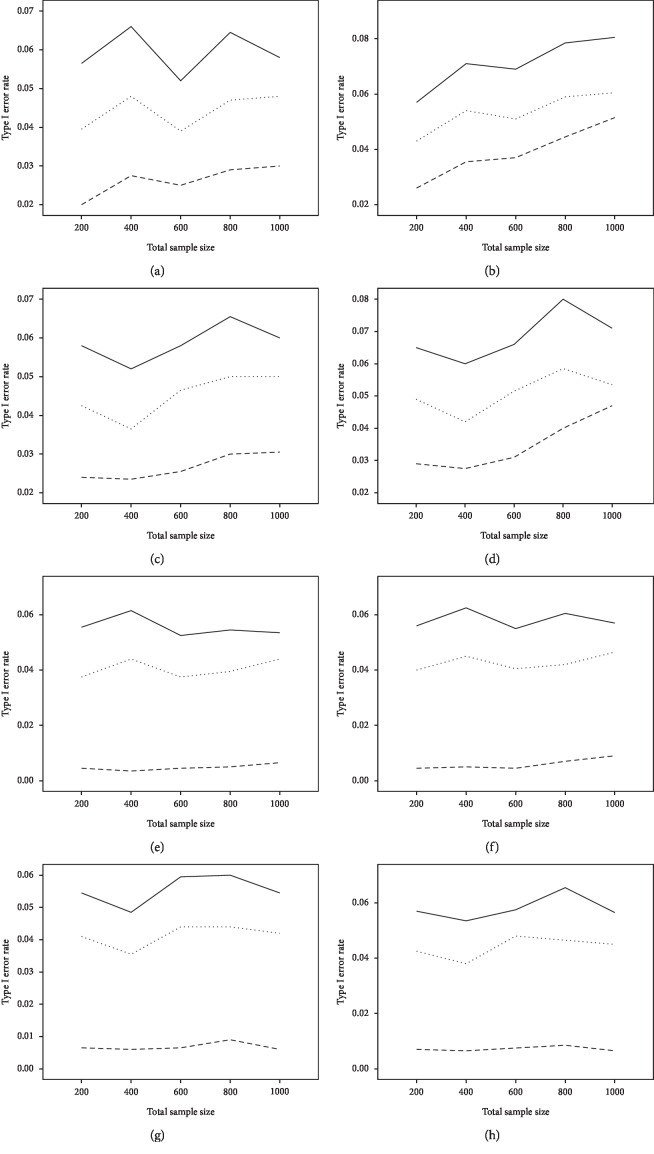
The average type I error rates of MLE (solid lines), PMLE (dotted line), and WLR (broken line) methods on measures with 5 and 15 items. *Note*. Left panel for DIF = 0.4 and right panel for DIF = 0.8. From top to bottom, the four panels are (*n*_f_ = *n*_r_, *τ* = 0.156), (*n*_f_ = 3*n*_r_, *τ* = 0.156), (*n*_f_ = *n*_r_, *τ* = 0.069), and (*n*_f_ = 3*n*_r_, *τ* = 0.069).

The average power and type I error rate on measures with 5 and 15 items are depicted in Figures 1 and 2. According to Figure 1, irrespective of the number of items, the highest power in all combinations belonged to WLR, while PML and ML had relatively equivalent power.  Figure 2 indicates that, in most simulation conditions, type I error rate was above or exactly at the nominal significance level of 0.05 for ML and below or exactly equal to the nominal level for the PML method (ML; range: 0.05–0.11and PML; range: 0.03–0.05). However, in WLR, type I error rate was lower than the other two methods and fluctuated between 0.02 and 0.05, from 0 to 0.01 for *τ* = 0.156 and *τ* = 0.069, respectively.

### 3.1. Real Data Example

In this section, we used a real data set to validate the simulation findings. Accordingly, 387 Serbian individuals (40.3% male and 50.7% female) were selected out of a larger sample of 4192 people from eleven countries participating in a cross-cultural study [31]. The participants completed the 47-item Revised Child Anxiety and Depression Scale (RCADS). The RCADS divided into six subscales including social phobia, separation anxiety disorder, generalized anxiety, panic disorder, obsessive compulsive disorder, and major depressive disorder. All items use the same 4-point Likert-type response scales to assess the frequency of a certain symptom. We binarized the item responses after transformation of never and sometimes into “no symptom,” and often and always into “symptom.” For details on the RCADS as used in this project, see [31]. The results of DIF analysis of the RCADS across male and female Serbian adolescents based on three inferential methods (WLR, PML, and ML) are shown in Table 3. Our findings showed that ten items with DIF and 33 items without DIF were common across the three methods. However, the results indicate that the WLR method is more sensitive than the PML and ML for detecting DIF. For example, in the separation anxiety subscale, while the WLR model identified four items with DIF, one and three items exhibited DIF according to PML and ML, respectively. Moreover, as compared with ML and PML, WLR gives smaller standard error for regression coefficient, and it can result in increased likelihood of finding DIF.

## 4. Discussion

Detecting DIF based on the logistic regression model can be challenging for binary data where events are rare or severely imbalanced. The present study is designed to clarify the unknown consequences of rare events data in the context of DIF analysis based on the LR model with and without applying bias correction methods. Hence, in the comprehensive simulation study, we compared the performance of the WLR, PML, and ML methods for detecting DIF with focus on imbalanced binary response data.

According to our simulation findings, of the three estimation methods for detecting DIF, the WLR seems to be the most appealing with regards to its statistical properties: its type I error rate is close to or less than the nominal 0.05 level and its power is considerably higher than the PML and ML. Moreover, in the present study, comparing the three inferential methods for the real data set confirmed the findings of the simulation. Accordingly, as compared with WLR, PML and ML were less sensitive for detecting DIF across male and female Serbian adolescents.

It is worthwhile to explain why the WLR outperforms the PML and ML estimation methods for DIF analysis. Indeed, the WLR is an extension of the small-sample bias corrections, as described by Cordeiro and McCullagh, to the weighted likelihood [32]. A previous simulation study has shown that this bias correction method reduces both the bias and the variance, thereby offering the smallest Mean Squared Error (MSE) when compared with that of PML and ML estimation methods [33]. Hence, applying the WLR in DIF analysis can lead to reduction in the standard error of the parameter estimates, which consequently increases the likelihood of finding DIF. Another advantage of the WLR is that it gives unbiased predicted probability [15, 20]. However, the predicted probabilities from the WLR model may fall outside the plausible range of 0 to 1 [15].

Furthermore, our findings revealed that the logistic regression model based on the traditional ML estimation method had a slightly better performance than the PML for imbalanced or rare events data. These findings were similar to those of Lee who reported that the ML slightly outperformed the PML for detecting DIF in small samples [9]. In this case, as described by Lee, it seems that the sacrifice in the precision cannot be compensated for the bias reduction of the PML estimates in imbalanced data. It should also be noted that while Firth's PML method reduces the bias in the estimates of regression coefficients, it introduces bias in the predicted probabilities, and this bias is not negligible for rare events data [15]. To overcome this problem, Puhr et al. proposed two modified versions of Firth's PML resulting in unbiased predicted probabilities [15]. Accordingly, the proposed methods, including Firth's logistic regression with intercept correction and Firth's logistic regression with added covariate, efficiently improve on predictions from Firth's logistic regression [15]. Since these methods were not implemented in the standard statistical software such as R, we were not able to apply them in the context of DIF analysis and compare their results with those of the WLR model. Hence, in the present study, the WLR is the most efficient method for DIF analysis under rare events data. The WLR not only reduces the bias of regression coefficients but also provides unbiased predicted probability in comparison to the PML method [15, 20].

What distinguishes this study from the previous one is that we simultaneously evaluated the effect of imbalanced data and small samples (as well as large samples) on the performance of the three estimation methods for DIF analysis. In addition, one of the advantages of the present study is that it provides a guideline about the required sample size for DIF analysis with imbalanced or rare events data. Previous simulation studies have shown that the minimum sample size for DIF analysis with logistic regression should be within the range of 100 to 200 per group [34, 35]. However, for moderate imbalanced data (*τ* = 0.156) and severe DIF (DIF = 0.8), as a general rule of thumb, we would suggest imposing a minimum of 300 respondents per group to achieve the adequate power with the WLR method. In addition, for severe imbalance rate (*τ* = 0.069) and DIF = 0.8, the WLR performs well to ensure the acceptable power with samples of 500 per group. To find what the minimum number of sample size is to achieve the adequate power (%80) for moderate DIF, we simulated items with DIF = 0.4, *τ* = 0.156 and 0.069, and sample sizes greater than 500 in each group, not reported in the results section. The findings revealed that sample sizes of at least 1000 per group for *τ* = 0.156 and 2000 per group for *τ* = 0.069 are required to detect DIF = 0.4 based on the WLR method.

In the present study, we restricted our simulation to only two bias correction methods for detecting DIF, namely, WLR and PML. Nevertheless, there are a limited number of studies that evaluated DIF based on other penalization methods such as LASSO. For example, Tutz and Schauberger applied LASSO penalization for DIF analysis which includes continuous variables [36]. Furthermore, Magis et al. proposed a new DIF detection method based on LASSO where all items were simultaneously evaluated for DIF in a single modeling approach so that multiple testing would not be a problem [37].

## 5. Conclusion

Although the logistic regression (LR) model is one the most common methods for detecting DIF, certain sampling strategies and appropriate bias correction techniques should be applied when LR is implemented on moderate or severe imbalanced data sets. In summary, our findings revealed that, as compared with ML and PML, the WLR is a more sensitive method for detecting DIF when data are imbalanced or rare. Hence, as well as its easy application to existing software, the WLR introduced by King and Zeng is strongly recommended for detecting DIF due to higher power and lower type I error rate in comparison to PML and ML inferential methods. However, in the future studies of DIF, penalized and bias correction methods should be proposed for ordinal logistic regression in the presence of rare events or small sample setting.

## Figures and Tables

**Table 1:** Statistical power of different methods of estimation under different combinations.

Item	Ratio	*N*	DIF: 0.4						DIF: 0.8					
			*τ*: 0.156			*τ*: 0.069			*τ*: 0.156			*τ*: 0.069		
			ML	PML	WLR	ML	PML	WLR	ML	PML	WLR	ML	PML	WLR
5	*n*_f_ = *n*_r_	200	0.13	0.10	0.16	0.09	0.06	0.12	0.34	0.30	0.42	0.20	0.15	0.28
		600	0.29	0.26	0.33	0.17	0.15	0.21	0.78	0.75	0.83	0.49	0.45	0.58
		1000	0.46	0.42	0.51	0.23	0.21	0.27	0.94	0.92	0.96	0.69	0.66	0.77

5	*n*_f_ = 2*n*_r_	200	0.13	0.11	0.16	0.09	0.07	0.13	0.32	0.29	0.41	0.19	0.15	0.30
		600	0.29	0.27	0.32	0.16	0.15	0.22	0.74	0.72	0.82	0.45	0.44	0.59
		1000	0.41	0.39	0.46	0.23	0.22	0.29	0.89	0.88	0.93	0.62	0.61	0.73

5	*n*_f_ = 3*n*_r_	200	0.13	0.11	0.17	0.09	0.08	0.16	0.30	0.28	0.41	0.17	0.16	0.29
		600	0.21	0.20	0.26	0.13	0.13	0.18	0.61	0.60	0.73	0.37	0.36	0.51
		1000	0.37	0.35	0.42	0.21	0.20	0.26	0.86	0.85	0.91	0.56	0.56	0.70

15	*n*_f_ = *n*_r_	200	0.13	0.11	0.17	0.10	0.09	0.13	0.36	0.33	0.45	0.22	0.18	0.29
		600	0.34	0.32	0.38	0.20	0.19	0.23	0.81	0.80	0.87	0.52	0.49	0.62
		1000	0.48	0.46	0.53	0.28	0.25	0.32	0.96	0.96	0.98	0.74	0.72	0.81

15	*n*_f_ = 2*n*_r_	200	0.13	0.12	0.17	0.11	0.09	0.15	0.35	0.33	0.44	0.22	0.20	0.32
		600	0.34	0.32	0.39	0.20	0.20	0.25	0.77	0.77	0.85	0.51	0.50	0.65
		1000	0.45	0.44	0.50	0.25	0.24	0.31	0.94	0.94	0.97	0.70	0.70	0.80

15	*n*_f_ = 3*n*_r_	200	0.13	0.13	0.17	0.10	0.08	0.14	0.31	0.31	0.41	0.19	0.19	0.31
		600	0.25	0.25	0.31	0.15	0.15	0.21	0.74	0.74	0.83	0.43	0.44	0.57
		1000	0.37	0.37	0.43	0.23	0.24	0.30	0.90	0.89	0.94	0.64	0.66	0.77

*Note*. Ratio: sample size ratio between the reference and focal groups. *n*_r_ and *n*_f_ represent sample sizes in reference and focal groups, respectively. *N* = total sample size; *N* = *n*_r_ + *n*_f_. *τ*: the fraction of 1s in the population. DIF: differential item functioning; ML: maximum likelihood; PML: penalized maximum likelihood; WLR: Weighted Logistic Regression.

**Table 2:** Type I error rate of different methods of estimation under different combinations.

Item	Ratio	*N*	DIF: 0.4						DIF: 0.8					
			*τ*: 0.156			*τ*: 0.069			*τ*: 0.156			*τ*: 0.069		
			ML	PML	WLR	ML	PML	WLR	ML	PML	WLR	ML	PML	WLR
5	*n*_f_ = *n*_r_	200	0.07	0.05	0.02	0.07	0.05	0.01^*∗*^	0.07	0.05	0.03	0.07	0.05	0.01^*∗*^
		600	0.05	0.04	0.03	0.05	0.03	0.01^*∗*^	0.08	0.06	0.05	0.06	0.04	0.01^*∗*^
		1000	0.07	0.05	0.04	0.06	0.04	0.01	0.11	0.08	0.08	0.06	0.05	0.01

5	*n*_f_ = 2*n*_r_	200	0.07	0.05	0.03	0.07	0.05	0.01	0.08	0.06	0.04	0.07	0.05	0.01
		600	0.06	0.04	0.02	0.04	0.04	0.01	0.08	0.06	0.05	0.05	0.04	0.01
		1000	0.06	0.05	0.03	0.05	0.04	0.01	0.11	0.09	0.07	0.06	0.04	0.01

5	*n*_f_ = 3*n*_r_	200	0.06	0.04	0.03	0.05	0.04	0.01	0.07	0.05	0.04	0.06	0.04	0.01
		600	0.07	0.05	0.03	0.07	0.05	0.01	0.08	0.06	0.04	0.07	0.05	0.01
		1000	0.07	0.06	0.04	0.06	0.04	0.01	0.09	0.07	0.07	0.06	0.05	0.01

15	*n*_f_ = *n*_r_	200	0.05	0.03	0.02	0.05	0.03	0.01	0.05	0.03	0.02	0.05	0.03	0.01
		600	0.05	0.04	0.02	0.05	0.04	0.01	0.06	0.04	0.03	0.05	0.04	0.01
		1000	0.05	0.05	0.03	0.05	0.05	0.01	0.05	0.05	0.03	0.05	0.05	0.01

15	*n*_f_ = 2*n*_r_	200	0.07	0.05	0.03	0.06	0.05	0.01^*∗*^	0.07	0.05	0.03	0.07	0.05	0.01^*∗*^
		600	0.06	0.04	0.03	0.06	0.05	0.01	0.06	0.05	0.03	0.06	0.05	0.01
		1000	0.04	0.04	0.02	0.04	0.03	0.01^*∗*^	0.05	0.04	0.02	0.04	0.04	0.01^*∗*^

15	*n*_f_ = 3*n*_r_	200	0.06	0.05	0.02	0.06	0.05	0.01	0.06	0.05	0.02	0.06	0.05	0.01
		600	0.05	0.05	0.02	0.05	0.04	0.01^*∗*^	0.05	0.05	0.02	0.05	0.05	0.01^*∗*^
		1000	0.05	0.04	0.02	0.05	0.04	0.01	0.05	0.04	0.02	0.05	0.04	0.01^*∗*^

*Note*. Ratio: sample size ratio between the reference and focal groups. *n*_r_ and *n*_f_ represent sample sizes in reference and focal groups, respectively. *N* = total sample size; *N* = *n*_r_ + *n*_f_. *τ*: the fraction of 1s in the population. DIF: differential item functioning; ML: maximum likelihood; PML: penalized maximum likelihood; WLR: Weighted Logistic Regression. ^*∗*^Near to 0.01.

**Table 3:** The results of DIF analysis across male and female Serbian individuals based on ML, PML, and WLR methods.

	Item	ML			PML			WLR			
		B	SE	*P* value	B	SE	*P* value	B	SE	*P* value	*τ*
Social phobia	7	0.61	0.29	0.032	0.6	0.29	0.042	0.61	0.31	0.042	0.24
	20	−1.16	0.44	0.007	−1.13	0.44	0.01	−1.19	0.42	0.002	0.17
	38	1.24	0.44	0.002	1.19	0.43	0.004	1.18	0.41	0.001	0.19
	43	−1.57	0.47	<0.001	−1.52	0.46	0.001	−1.56	0.44	<0.001	0.19

Separation anxiety	9	−0.89	0.48	0.06	−0.86	0.47	0.1	−0.83	0.36	0.015	0.31
	17	2.06	1.22	0.045	1.7	1.06	0.092	0.97	0.75	0.008	0.11
	45	0.96	0.48	0.034	0.91	0.47	0.059	1.04	0.56	0.036	0.09
	46	−3.04	1.14	0.001	−2.69	1.01	0.003	−1.9	0.51	<0.001	0.14

Generalized anxiety disorder	13	1.14	0.35	0.001	1.12	0.35	0.001	1.12	0.35	0.001	0.31
	37	−2.01	0.44	<0.001	−1.96	0.44	<0.001	−1.95	0.44	<0.001	0.22

Obsessive compulsive disorder	23	0.79	0.43	0.059	0.75	0.43	0.1	0.79	0.41	0.038	0.18

Major depression	2	1.23	0.45	0.004	1.18	0.44	0.006	1.19	0.45	0.003	0.19
	6	−1.22	0.58	0.031	−1.18	0.56	0.043	−1.15	0.42	0.004	0.12
	21	0.63	0.32	0.048	0.62	0.32	0.072	0.65	0.34	0.049	0.26

*Note*. *P* value is reported in three decimal places for more accuracy of comparing the three models. *τ*: the fraction of 1s in the population that is extracted from a data set of 4192 adolescents in eleven countries. B: regression coefficient for testing uniform DIF. SE: standard error of regression coefficient.

## Data Availability

This is a simulation study, and the real data set has been provided by Dr. Dejan Stevanovic the third author of the manuscript.
